# A comparative analysis of centralized waiting lists for patients without a primary care provider implemented in six Canadian provinces: study protocol

**DOI:** 10.1186/s12913-017-2007-8

**Published:** 2017-01-21

**Authors:** Mylaine Breton, Michael Green, Sara Kreindler, Jason Sutherland, Jalila Jbilou, Sabrina T. Wong, Jay Shaw, Valorie A. Crooks, Damien Contandriopoulos, Mélanie Ann Smithman, Astrid Brousselle

**Affiliations:** 1 Charles-LeMoyne Hospital Research Centre, Sherbrooke University, Longueuil Campus, 150 Place Charles-LeMoyne, Office 200, Longueuil, QC J4K 0A8 Canada; 2 Family Medicine and Public Health Sciences and CHSPR, Queen’s University, Abramsky Hall, 3rd Floor 21 Arch St., Kingston, ON K7L 3N6 Canada; 3 Manitoba Research Chair in Health System Innovation and Community Health Sciences, University of Manitoba, 200-1155 Concordia Ave., Winnipeg, MB R2K 2M9 Canada; 4 Centre for Health Services and Policy Research, University of British Columbia, 201-2206 East Mall, Vancouver, BC V6T 1Z3 Canada; 5 School of psychology, Université de Moncton, Centre de formation médicale du Nouveau-Brunswick, Pavillon Léopold-Taillon Université de Moncton, 18 Ave Antonine-Maillet, Moncton, NB E1A 3E9 Canada; 6 School of Nursing and Centre for Health Services and Policy Research in the School of Population and Public Health, University of British Columbia, 2211 Wesbrook Mall, Vancouver, BC V6T 2B5 Canada; 7 Institute for Health System Solutions and Virtual Care, Women’s College Hospital, 76 Grenville Street, Toronto, ON M5S1B2 Canada; 8 Canada Research Chair in Health Service Geographies, Simon Fraser University, 8888 University Drive, Burnaby, BC V5A 1S6 Canada; 9 Faculty of nursing, University of Montréal, 2375, chemin de la Côte-Ste-Catherine, Montréal, Québec H3T 1A8 Canada

**Keywords:** Primary health care, Health services accessibility, Comparative study, Waiting lists, Physicians, General practitioners, Physician patient relationship, Unattached patients

## Abstract

**Background:**

Having a regular primary care provider (i.e., family physician or nurse practitioner) is widely considered to be a prerequisite for obtaining healthcare that is timely, accessible, continuous, comprehensive, and well-coordinated with other parts of the healthcare system. Yet, 4.6 million Canadians, approximately 15% of Canada’s population, are unattached; that is, they do not have a regular primary care provider. To address the critical need for attachment, especially for more vulnerable patients, six Canadian provinces have implemented centralized waiting lists for unattached patients. These waiting lists centralize unattached patients’ requests for a primary care provider in a given territory and match patients with providers. From the little information we have on each province’s centralized waiting list, we know the way they work varies significantly from province to province. The main objective of this study is to compare the different models of centralized waiting lists for unattached patients implemented in six provinces of Canada to each other and to available scientific knowledge to make recommendations on ways to improve their design in an effort to increase attachment of patients to a primary care provider.

**Methods:**

A logic analysis approach developed in three steps will be used. Step 1: build logic models that describe each province’s centralized waiting list through interviews with key stakeholders in each province; step 2: develop a conceptual framework, separate from the provincially informed logic models, that identifies key characteristics of centralized waiting lists for unattached patients and factors influencing their implementation through a literature review and interviews with experts; step 3: compare the logic models to the conceptual framework to make recommendations to improve centralized waiting lists in different provinces during a pan Canadian face-to-face exchange with decision-makers, clinicians and researchers.

**Discussion:**

This study is based on an inter-provincial learning exchange approach where we propose to compare centralized waiting lists and analyze variations in strategies used to increase attachment to a regular primary care provider. Fostering inter-provincial healthcare systems connectivity to improve centralized waiting lists’ practices across Canada can lever attachment to a regular provider for timely access to continuous, comprehensive and coordinated healthcare for all Canadians and particular for those who are vulnerable.

**Electronic supplementary material:**

The online version of this article (doi:10.1186/s12913-017-2007-8) contains supplementary material, which is available to authorized users.

## Background

Attachment, a formal or informal affiliation to a regular provider such as a nurse practitioner or family physician [1], is widely considered to be a prerequisite for primary healthcare that is accessible, continuous, comprehensive, and well-coordinated with other levels (e.g., secondary, tertiary) or types (e.g., social, community-based) of care [2–14]. There is strong evidence in the scientific literature that suggests that patients who are attached to a primary care provider benefit from better care coordination [15–17], chronic disease management [18, 19] and receive more preventative care [20, 21], use emergency services less frequently [22–24] and have better health outcomes [25, 26].

Compared to other OECD countries, Canada ranks low in terms of access to a primary care provider [27]. Thus, attachment to a primary care provider is an especially important issue in Canada because this is most often the first point of contact to the rest of the healthcare system. For example, patients need a referral from a family physician or need to go through the emergency department to access specialized care. Canadian primary care providers’ role goes beyond providing primary healthcare and preventive care [28] as the structure of healthcare systems positions them as the “gatekeepers” to secondary and tertiary care [29–31]. Access to healthcare for unattached patient is therefore limited in Canada [32].

Several Canadian commissions on healthcare have recommended that primary healthcare be reinforced to guarantee access to a primary care provider to every Canadian [33–38]. Yet, 4.6 million Canadians, approximately 15% of Canada’s population, do not have a regular primary care provider [39]. Moreover, in Canada, vulnerable patients, those with multiple intersecting determinants of health including complex physical and mental health and healthcare needs, being young or a recent immigrant, having a low income level, living in a rural or remote area, and those with low social support are less likely to be attached to a primary care provider [40, 41] despite being the ones who would benefit most from access to comprehensive and continuous primary care [42, 43].

### Centralized waiting lists for unattached patients

To address the critical need for attachment, six provinces have implemented centralized waiting lists to coordinate the supply of primary care providers and demand of patients for attachment (see Table 1). These waiting lists generally aim to centralize unattached patients’ requests for a primary care provider in a given jurisdiction and to match unattached patients with providers, based on availability of primary care workforce and, in some cases, medical need [32, 44].

**Table 1:** Centralized waiting lists for unattached patients implemented across Canada

Province	Program Name	Implementation Year
Prince Edward Island	Patient Registry Program	1998
Quebec	*Guichets d’accès à un médecin de famille*	2008
Ontario	Health Care Connect	2009
Manitoba	Family Doctor Finder	2013
New-Brunswick	Patient Connect NB	2013
British Columbia	A GP for Me	2015

In Canada, the provinces and territories administer and deliver most health care services [45]. The roles of the provinces/territories include administration of their health insurance plans; planning and funding of services in hospitals and other health facilities as well as of services provided by doctors and other health professionals and planning and implementing health promotion and public health initiatives. Therefore, initiatives such as centralized waiting lists for unattached patients may be quite different in each province or territory. Moreover, there is limited knowledge exchange between provinces regarding such initiatives.

### Wide variations in centralized waiting lists for unattached patients across Canada

From the little information available, we know there are wide variations between provinces in the way centralized waiting lists for unattached patients work [46]. Each province’s waiting list has distinct characteristics. This study will lead to a better understanding of the components of each of the six centralized waiting lists. A few examples of variations are described below.

#### Guidelines for the centralized waiting lists

**Patient eligibility** for registering in a centralized waiting list differs from one province to another. In Ontario, patients who already have a primary care provider are not eligible to register in the centralized waiting list, while in New Brunswick, patients who have a provider, but wish to change providers are allowed to register on the list [47, 48]. The level of **governance** of centralized waiting lists is also variable. In Ontario, the program is managed at a provincial level with a delegation of management to the Community Care Access Centers at a regional level; while in Quebec, the program is managed at a local level by the Integrated Centers for Health and Social Services with some provincial oversight.

#### Incentives to providers to attach new patients

Incentives to providers to attach new patient differ from one province to another. In Quebec, primary care providers receive a one-time **financial incentive** to attach a new patient. These incentives are more substantial if the patient is attached via a centralized waiting list. For these patients, the presence of specific types of vulnerabilities determines the amount physicians will receive: CAD $23 to attach a “non-vulnerable patient” (i.e., healthy patient), CAD$150 to attach a “vulnerable patient” (i.e., with at least one chronic disease or over 70 years old), and CAD$300 to attach a “super vulnerable patient” (i.e., suffering from mental illness or substance abuse) [49]. In Ontario, family physicians receive a one-time payment of CAD$350 for attaching a new “complex-vulnerable patient” from the centralized waiting list [50].

#### Structure to receive & follow-up requests for a provider from patients

The structure for receiving requests for primary care providers varies quite a bit between provinces. In New Brunswick, for example, unattached patients contact Tele-care 8-1-1, a pre-existing health advice and information line, to register on the centralized waiting list [51]. In Quebec, requests for a family physician can be made through a website hosted by the provincial health insurance. Additionally, each local health network has a nurse and administrative assistant who are available to assist patients fill out a request [44, 52]. In Ontario and Manitoba, this part of the process is outsourced to a third party.

#### Identifying & prioritising vulnerable patients

Moreover, there seems to be significant differences in how **vulnerable patients** are defined and how they are **prioritized** when being attached to a primary care provider. In Quebec, patients are prioritized into five priority categories (A to E) at a local level based on the urgency of their need for a primary care provider [52, 53]. The urgency of need is assessed based on the self-reported presence of specific types of vulnerabilities (e.g., diabetes, mental illness, cancer, HIV/AIDs) or being over 70 years old. Patients may also request a health assessment by a nurse, which is done over the phone. There are provincially recommended wait times for each priority category: ≤7 days for A, ≤14 days for B, ≤21 days for C, ≤1 month for D and ≤3 months for E. In Ontario, “complex-vulnerable patients” are defined as having one or more co-morbidities, or being frail based on self-assessed health status, chronic conditions or health problems, activity limiting disability, mental health status and body mass index [54]. Priority is then given to those with the greatest need for a primary care provider. In New Brunswick, patients are asked to answer a health screening questionnaire regarding long term health conditions and need for follow-up (multiple medications, history of mental illness, children under 5, etc.), but patients are assigned to a primary care provider on a first-come, first serve basis [48].

#### Matching patients to providers

The way patients are matched with providers is different in each province. In Manitoba, patients are asked about their **provider preferences** (type of provider, language spoken by provider, provider gender, and distance they are willing to travel) [55]. Conversely, in Quebec, no questions are asked about patients’ preferences, the only consideration is geographic distance to the clinic [52, 56]. In addition, provinces such as Ontario and Manitoba offer attachment to a **nurse practitioner or family physician**, while Quebec, for instance, only offers attachment to family physicians. Provinces also have varying **processes** in place to match providers and patients. In Ontario, nurses called Care Connectors are locally mandated to help patients find a primary care provider and are the patients’ main contact during their time on the waiting list [57]. In Quebec, physicians called Local Medical Coordinators are mandated to help the centralized waiting list attach patients, but are not assigned to patients like in Ontario.

#### Impacts of centralized waiting lists

Currently, we have access to very little information that allows us to compare the impacts of centralized waiting lists across provinces. However, we know that the **volume of patients attached** to primary care providers varies significantly. For instance, in Manitoba, around 30 000 patients have been attached through Family Doctor Finder since its implementation in 2013 [58]. In comparison, the *guichets d’accès aux médecins de famille* in Quebec has attached over 800 000 patients since its implementation in 2008 [53]. We also know that, over the last year, the effectiveness of the centralized waiting list in **attaching complex patients** has been heterogeneous. Ontario has successfully attached 85% of what they identified as being “complex-vulnerable patients” [59]; while Quebec has attached only 20% of the patients identified as being “vulnerable”.

#### British Columbia: a special case

Most centralized waiting lists in the other five provinces were mandated at a provincial level and implemented more or less uniformly across the province with slight variations in local practices. However, for the “A GP for Me” initiative in British Columbia, each division analyzed their needs and implemented various strategies to improve attachment according to those needs. Therefore, only a handful of divisions implemented some type of centralized waiting list for unattached patients. The case of British Columbia, although different from the other provinces, is especially interesting because of the variety of innovative strategies combined with centralized waiting lists to improve attachment. Therefore, we will identify common characteristics at a provincial level, but will also conduct an in-depth analysis of some divisions.

### Study objective

The main objective of this study is to compare the different models of centralized waiting lists for unattached patients implemented in six provinces of Canada to each other and to available scientific knowledge to make recommendations on ways to improve their design in an effort to increase attachment of patients to a primary care provider.

## Methods

### Overall study design

We will conduct a logic analysis to compare, analyze and identify improvement strategies for the centralized waiting lists for unattached patients across Canada. All six provinces that have implemented a centralized waiting list for unattached and complex patients will be included in this study.

Logic analysis is a theory-based evaluation that tests the adequacy between the intended outcomes of an intervention and the actions undertaken to achieve those outcomes [60, 61]. It assesses the validity of the intervention’s theory by identifying the main characteristics of the program and the key contextual factors that may affect the intervention’s effectiveness to produce intended outcomes [61, 62].

There are several benefits of using a logic analysis to evaluate an intervention. First, because it examines the fit between actual activities and strategies of the intervention, and those that should be implemented to achieve the intended outcomes, the use of a logic analysis may reveal gaps in logic [63]. Second, testing the program’s theory can provide important insights into the intervention’s strengths and weaknesses while mobilizing various stakeholders in a valuable reflection process [60]. It can therefore provide stakeholders with a rapid feedback on the validity of their intervention and on the ways they can improve the effectiveness of their intervention. Finally, it allows for the identification of adequate performance indicators based on data that is relevant to the intervention’s components.

A logic analysis is conducted in three steps: 1) building logic models of the interventions, 2) developing a conceptual framework based on scientific knowledge (literature and expert views), and 3) comparing the logic models to the conceptual framework [60].

#### Step 1: Build logic models that describes each province’s centralized waiting list

The logic models will be elaborated based on Mitchell & Lewis’s *Manual to Guide the Development of Local Evaluation Plans* [63]. This particular model offers a simple diagram of the main components of a unique intervention and is widely used in research on primary healthcare interventions in Canada. For instance, Mitchell & Lewis’ model is currently being used by the 12 research programs in the Canadian Institutes of Health Research's signature initiative on community based primary healthcare [64]. It is therefore relevant to use this model to evaluate complex, multilevel and multifaceted interventions such as centralized waiting lists for unattached patients.

This model assumes that inputs/strategies of the intervention will influence the *processes and structures*, which will then influence the *impacts*, which will in turn lead to the intended outcomes of the intervention. It aims to identify every component involved in the desired change, indicators to measure these components, as well as gaps in the intervention’s logic. Six categories of components must be included in the model.

- **Action areas**: the focus area of the intervention;
- **Outcomes**: intended changes in health and wellbeing of targeted population;
- **Inputs/strategies**: resources, strategies and activities needed to launch the intervention;
- **Processes and structures**: mechanisms and characteristics of services, systems or activities that have to be maintained over time to achieve impacts;
- **Impacts**: changes that are crucial to achieving intended outcomes;
- **Contextual factors**: political, cultural, socioeconomic and geographic factors that might affect the intervention’s effectiveness in producing intended outcomes.

**Fig. 1:**
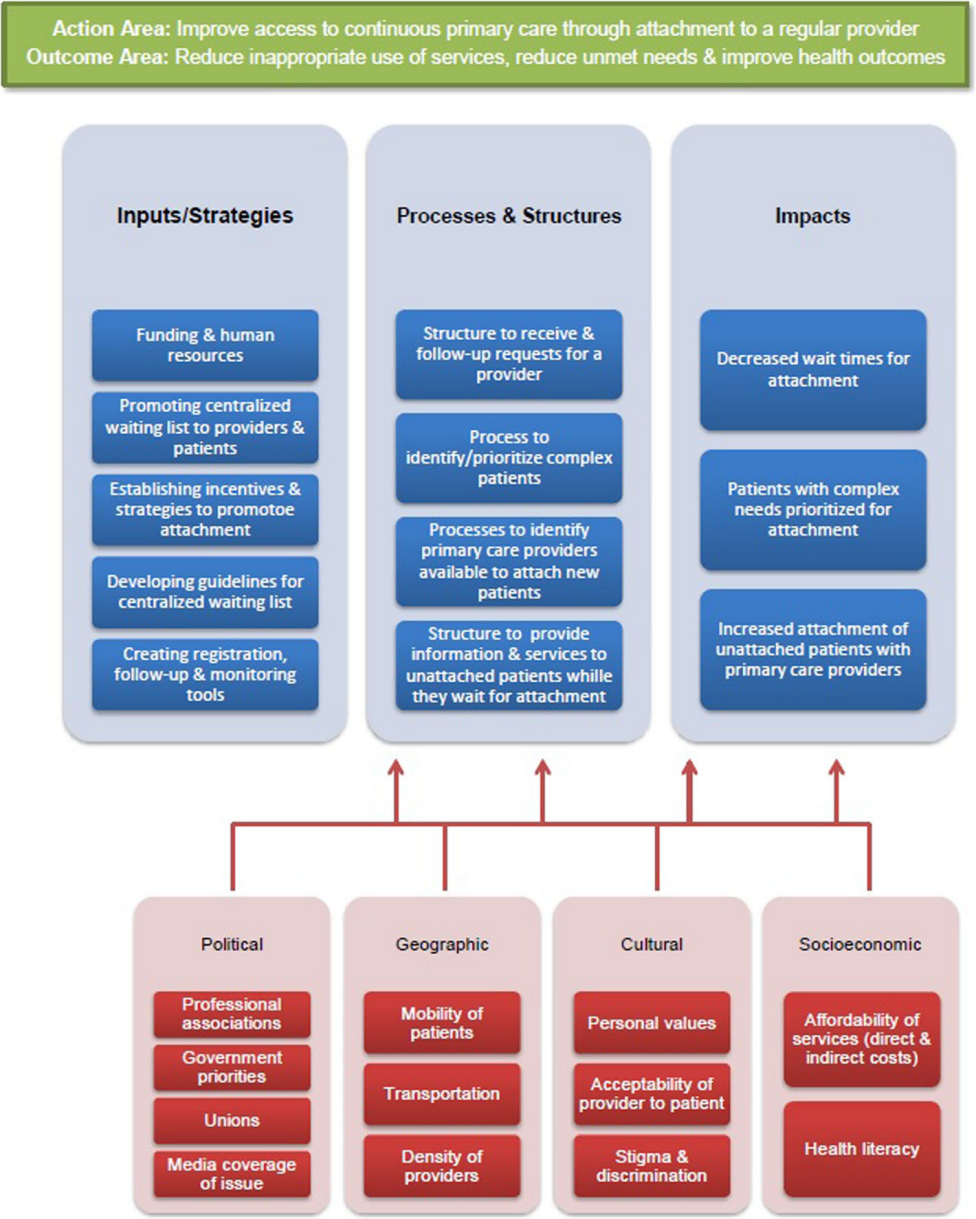
Logic model template

We have provided below an example of how the logic model will be used to map out the different components of centralized waiting lists for unattached patients (see Fig. 1).

In each of the six cases, a variety of data sources will be used. First, we will review the grey literature to identify the main characteristics of each centralized waiting list. The grey literature will be reviewed using search engines such as Google and Google Scholar as well as specific sources such as websites from health institutions, national and provincial government, provincial and local newspapers [65]. We will also use the grey literature to identify key stakeholders in each province (professional associations, decision-makers, clinicians, etc.). In addition, our knowledge users will help us identify key stakeholders of the centralized waiting list in their province. We will then conduct semi-structured interviews with the key stakeholders identified and use a snowballing method to identify additional participants. The aim of these interviews is to gain an in depth understanding of the characteristics each centralized waiting list (for more details, please consult interview guide in Additional file 1). Interviews will be conducted in person, by phone or by videoconference depending on the location of the interviewee. We plan to conduct interviews in each province until we reach saturation (around eight interviews per case). All interviews will be taped and transcribed when formally authorized by respondents.

All documents and interview transcriptions will be coded using NVivo (QSR) software. A list of initial codes based on the logic model template (Fig. 1) has been established. This list details the five elements of logical analysis on centralized waiting list which are 1) the needed inputs for implementation of centralized waiting list, 2) process for requesting a family physician, 3) prioritization of patients and attachment to a primary care provider, 4) perceived outputs of the centralized waiting list and 5) elements from the contexts that might have an influence on the implementation of the centralized waiting list. This list will be modified and enhanced over the course of the analyses.

Two independent team members will code the data to ensure reproducibility and reliability. A double-coding technique will be used in order to control the coding step and to guaranty the repeatability and reliability of the method. Coding results will be compared. The codes will be refined where differences appear and then coding will be repeated. This process will be repeated until the two analysts obtain inter-coder agreement of more than 90% [66]. Codes will be grouped in the corresponding themes and also in new themes that have appeared during analysis. We will analyze transcripts for each province separately in order to better understand the how each centralized waiting list works. Coded materials will be analyzed using a thematic analysis and results will be synthetized using tables and matrices [66, 67]. Data obtained for each province will be summarized.

The characteristics of each province’s centralized waiting list will be presented in a logic model. We will then use these logic models to perform a cross-case (inter-provincial) analysis of the six centralized waiting lists. We will compare the centralized waiting lists, highlight similarities and differences between them and identify factors that can lead to an increase in attachment of patients to a primary care provider. The logic models will be presented in a graphic form. We will collaborate closely with a graphic designer to ensure the models are easy to understand. The information in each logic model will be validated by key stakeholders from the centralized waiting list in each province. A face-to-face meeting will be organized on each site between the key informants and members from our team.

#### Step 2: Develop a conceptual framework based on key characteristics of centralized waiting lists for unattached patients and factors influencing their implementation

Developing a conceptual framework is a core component of the logic analysis and critical to assessing an intervention’s rationale. This step consists of identifying “the best ways of doing things” [60]. This is done by analysing the centralized waiting lists’ components and identifying the optimal way to achieve the intended outcomes or by identifying alternative ways, if any, of achieving these outcomes.

In order to develop a conceptual framework, **a realist review** approach will be used. Based on evidence from the scientific literature and experts’ views related to the intervention [61, 68, 69], the realist review is a theory-driven approach developed in order to explore causal processes that generate outcomes within programs or interventions. This approach aims to evaluate the context, mechanism and outcomes of interventions with a heterogeneous body of evidence. Given that this study is being conducted over a 12 month period and that there is very limited literature on centralized waiting lists for unattached patients, this approach was chosen in order to assemble a body of evidence on the mechanisms of different aspects of centralized waiting lists for unattached patients.

We will use the realist review approach for three key components of centralized waiting lists for unattached patients:

1. What are unattached patients’ characteristics and primary care needs?
2. What are the best ways to manage centralized waiting lists?
3. What are the most effective incentives (financial or other) to increase the number of new patients attached to primary care providers?

The realist review will comprise three distinct review processes, covering the three aforementioned aspects. The primary literature searches will be performed by a member of the research team with the EBSCOhost interface through four databases: Medline, CINAHL, PsychInfo and SocIndex. The research will be limited to peer-reviewed texts in French and English published between 2000 and 2016. A Boolean search will be performed using search terms specific to each subject developed by the research team with the help of an information specialist.

A classic two-step review selection process will be conducted by two reviewers to identify the relevant articles to be included in the review. The first step of the selection process will be a review based on articles' title and abstract. Articles from the primary literature research will be reviewed by the two reviewers on the basis of potential relevance regarding the process of each research questions. Following the title and abstract screening, articles will be screened for full text review by the two reviewers based on agreement of reviewing and discussion on disagreement. Criteria for the full text review will be elaborated by main researcher and the reviewers in order to proceed to the selection of the relevant texts to be included on the studies.

To ensure the maximal selection of relevant articles, each of the selected will get their references screened and a research through Google Scholar will be done to find articles citing the article as well as similar articles. An expert in each of components studied in this review will also be contacted in order to validate the content of the selected data and ensure that important articles were not forgotten in the process.

Data will be extracted following an extraction grid developed by a member of the research team for each of the three research aspects. A second member of the research team will review the extraction to ensure all relevant information has been extracted. The research team will construct the extraction grids in order to extract all relevant information that can be linked to the research questions. The analysis of the data collected with the extraction grids will be done following a thematic analysis method.

To ensure balance between relevance and scientific rigour, papers will be assessed based on both relevance and quality. The quality appraisal will be based on the prompts outlined by Dixon & Woods [70] and will be used to determine the contribution of the data to the reviews.

#### Step 3: Compare the logic models to the conceptual framework to make recommendations to improve centralized waiting lists in different provinces

We will compare the logic models built in step 1 to the conceptual framework developed by consulting experts and reviewing the literature in step 2. This should produce new readings of the centralized waiting lists for unattached patients that highlight strengths and weaknesses, as well as the strength of the causal chain toward the impacts and intended outcomes [60]. Our aim is to identify the strengths and weaknesses of each centralized waiting list under study, the characteristics that seem promising to promote attachment, particularly for vulnerable patients and the contextual factors that may affect the intervention’s effectiveness in order to propose strategies to improve attachment to a primary care provider that are relevant to each province’s context.

This step will be done using a participatory approach during a one-day face-to-face meeting with the research team and key stakeholders involved in centralized waiting lists for unattached patients across the six provinces. A participative approach will create consensus on what is required to improve current practices and adapt promising strategies to different contexts, enhance the appropriation of results, and initiate the necessary changes [71].

### Results’ dissemination

Promising strategies will be described in short videotaped interviews with the decision-makers of each province. The footage from these interviews will be used to put together short video clips. The video editing will be done in the multimedia laboratory at the Centre de recherche Hôpital Charles-Le Moyne. Also, the promising strategies will be summarized in a brief report. A dissemination strategy of the video clips, logic model illustrations and brief report produced will be developed in complementarity to the production of peer-reviewed scientific articles and presentations in international conferences.

## Discussion

No study has compared these complex organizational models across Canada and analyzed variations in strategies used to increase attachment to a regular primary care provider, for the general population and for vulnerable populations. To our knowledge, only one study has examined the implementation and outcomes of centralized waiting lists for unattached patients and this study focuses on the province of Quebec [7] and further comparative studies are needed. The natural experimentation of centralized waiting lists for unattached patients implemented in six provinces represents a unique opportunity to better understand these different models, to learn from the different experiences and to identify promising strategies to improve the effectiveness of these centralized waiting lists. The results from this study will be useful for decision-makers of healthcare systems in Canada and countries with similar healthcare systems by providing strategies and key elements for implementing effective centralized waiting lists.

### Implications for implementation

This study is based on an inter-provincial learning exchange approach where we propose to compare centralized waiting lists and analyze variations in strategies used to increase attachment to a regular primary care provider. To date, there has been very little collaboration between the decision-makers of each province’s centralized waiting lists to discuss best practices or promising strategies. Our research team has developed collaborations with key stakeholders in each province of the six provinces that have implemented a centralized waiting list for unattached patients. Comparing the six provinces’ models to the conceptual framework will allow us to understand the differences between the centralized waiting lists in the six provinces, to explain these differences according to context, and identify strategies to improve certain components and mechanisms for the six centralized waiting lists to better achieve intended outcomes. Our inter-provincial learning exchange approach will allow stakeholders from different provinces to come together to discuss the results of our study which in turn will enhance their appropriation of results, and initiate conversations about changes that could be made in each province. In addition to our integrated approach with key stakeholders, six Strategy for Patient-Oriented Research (SPOR) on Primary and Integrated Health Care Innovation Networks have agreed to provide support in connecting with additional stakeholders and disseminating results of the study within their province.

Fostering inter-provincial healthcare systems' connectivity to improve centralized waiting lists’ practices across Canada can therefore lever attachment to a regular provider for timely access to continuous, comprehensive and coordinated healthcare for all Canadians and particular for those who are vulnerable.

## Additional files

Additional file 1: Semi-structured interview guide. Interview guide. (PDF 183 kb)
