# Supplementary material for: A comparative analysis of centralized waiting lists for patients without a primary care provider implemented in six Canadian provinces: study protocol
Source: BMC Health Serv Res. 2017 Jan 21;17:60. doi: 10.1186/s12913-017-2007-8 (PMC5251310; doi:10.1186/s12913-017-2007-8)
Supplement: Additional file 1: — Semi-structured interview guide. Interview guide. (PDF 183 kb) [file 12913_2017_2007_MOESM1_ESM.pdf]

Semi-structured interview guide

Action area, outcome areas

1) What are the objectives of your province's centralized waiting list for unattached patient?
2) Why was this mechanism implemented in your province?
   → Who were the leaders in developing the centralized waiting list?
   → Were you inspired from another province's waiting list?
   → What were the roles of the medical/professional federations in the implementation of centralized waiting lists?
   → Are there specific characteristics that set your province apart, a particular history, etc., and that have influenced the implementation of the centralized waiting list for unattached patients?

Inputs/Strategies

3) How is operating the waiting list?
   → What are the resources invested?
   → Who finances this program?
   → What are the governance structures of the centralized waiting list?
   → If there are multiple waiting lists in the province, how are they coordinated?

4) How is the waiting list publicized to the population and to the family physicians/nurse practitioner?

Processes /Structures

5) Can you describe the general process from registration on the centralized waiting list to attachment to a primary care provider? What are the main steps?
   → Is there any assistance provided to patients facing barriers to registering on the list?
   → Is the process the same for nurses practitioners as for family physicians?
   → What criteria is attachment based on? (geographical distance, provider's expertise, etc.)
   → Are there mechanisms in place to evaluate patients' vulnerabilities/medical needs? How are patients prioritized? How are vulnerable/complex patients defined?
   → Are there mechanisms in place if the attachment does not suit the patient or provider?

6) What are the services or the information offered to patients waiting for attachment on the centralized waiting list?

7) How does primary care provider’s participation in the centralized waiting list work?
→ What information is sent to the provider once they have accepted to attach a new patient? How is this information sent?

8) In your opinion, what are the factors that encourage providers to attach patients from the centralized waiting list? What are the barriers?

9) Once the patient is attached to a provider, is there a follow-up with these patients from the centralized waiting list mechanism?

Context

10) In your opinion, are there certain characteristics of the models of primary care in your province that influence the participation of provider in the centralized waiting list?
→ Are there financial incentives to encourage them to attach patients from the list?

Impacts

11) Finally, in your opinion what have been the impacts of the centralized waiting lists since their implementation?

12) Do you have tools to monitor the performance of the centralized waiting list? Who is in charge of monitoring? What information is used for monitoring? How is this information collected?

13) What do you think the average wait times are for attachment via the centralized waiting lists? Are there types of patients that wait longer/shorter periods?

14) Has the centralized waiting list had unexpected/adverse effects?

15) What would be some changes that could be implemented in your province to improve centralized waiting lists?
